# Effects of 17β-Estradiol on Monocyte/Macrophage Response to *Staphylococcus aureus*: An *In Vitro* Study

**DOI:** 10.3389/fcimb.2021.701391

**Published:** 2021-07-14

**Authors:** Clarissa Leal Silva e Souza, Camila Dutra Barbosa, Hanna I. L. N. Coelho, Manoel N. Santos Júnior, Elaine Novaes Barbosa, Éllunny Chaves Queiroz, Mauro Fernandes Teles, Déborah Cruz dos Santos, Rafaela Souza Bittencourt, Telma de Jesus Soares, Márcio Vasconcelos Oliveira, Jorge Timenetsky, Guilherme Barreto Campos, Lucas Miranda Marques

**Affiliations:** ^1^ Multidisciplinary Institute of Health, Federal University of Bahia (UFBA), Vitória da Conquista, Brazil; ^2^ Santo Agostinho School of Health (FASA), Santo Agostinho Colleges, Afya Educational, Vitória da Conquista, Brazil; ^3^ University of Santa Cruz (UESC), Ilhéus, Brazil; ^4^ Department of Microbiology, Institute of Biomedical Science, University of São Paulo, São Paulo, Brazil

**Keywords:** sex steroids, macrophage, immune response, immunomodulation, *Staphylococcus aureus*

## Abstract

To describe how 17β-estradiol (E2) influence in the monocyte/macrophage response induced by *S. aureus* in *in vitro* models of murine peritoneal macrophages (MPMs) and human peripheral blood monocytes (HPBM). MPMs (2 x 10^5^/ml) were isolated from sham (n=3) and ovariectomized (OVX) females (n = 3) and males (n = 3) after induction by thioglycolate. The MPMs obtained from OVX females and males were treated for 24 hours with 17β-estradiol (E2) (10^-7^ M), and after that, inoculation with *S. aureus* was carried out for 6 hours. The macrophages were collected and destined to evaluate the relative gene expression of TNF-α, IL-1β, IL-6, IL-8 and TLR2. For the *in vitro* model of HPBMs, six men and six women of childbearing age were selected and HPBMs were isolated from samples of the volunteers’ peripheral blood. In women, blood was collected both during menstruation and in the periovulatory period. HPBMs were inoculated with *S. aureus* for 6 hours and the supernatant was collected for analysis of cytokines by Luminex and the HPBMs were removed for analysis of 84 genes involved in the host’s response to bacterial infections by RT-PCR array. Previous treatment with E2 decreased the gene expression and production of proinflammatory cytokines, such as TNF-α, IL-1β and IL-6 and decreased the expression of TLR2 tanto em MPMs quanto em HPBMs. The analysis of gene expression shows that E2 inhibited the NFκB pathway. It is suggested that 17β-estradiol acts as an immunoprotective in the monocyte/macrophage response induced by *S. aureus*.

## Introduction

Sex steroids, specifically testosterone, estrogen and progesterone, are found in different concentrations between the sexes, with males typically having higher levels of testosterone and females having higher levels of estrogen and progesterone at reproductive ages (vom Steeg and Klein, 2016). Generally, testosterone has an immunosuppressive effect, while estrogen has an immunostimulating effect (Taneja, 2018). Sexual differences in immune responses have been observed in several species, ranging from insects to mammals, and in all of these species, innate and adaptive immune responses are typically less intense in men than in women (Klein and Flanagan, 2016). Thus, the frequency and severity of infectious diseases clearly varies between men and women. In general, men are more susceptible to gastrointestinal, respiratory and sepsis bacterial diseases, while women are more susceptible to bacterial infections of the genitourinary tract. However, these incidences depend on the population assessed, the animal model, and bacterial species (Klein et al., 2011; Vázquez-Martínez et al., 2018).

*Staphylococcus aureus* is a gram-positive bacteria that frequently and asymptomatically colonizes the anterior nostrils of humans and animals (Mehraj et al., 2016). Despite its commensal characteristics, *S. aureus* is a frequent cause of clinically important and life-threatening infections (Wertheim et al., 2005; Mcguinness et al., 2017). Scientific evidence pointing to the hormonal influence on infections caused by *S. aureus* dates back to the 1960s (Yotis, 1967) and, in general, antimicrobial and anti-inflammatory effects of estradiol have been pointed out in infections mediated by *S. aureus* (Fahey et al., 2008; Gjertsson et al., 2012; Medina-Estrada et al., 2016; Kuwahara et al., 2017). However, data in the literature are still controversial, since being female was significantly associated with nosocomial bacteremia caused by MRSA (Vidal et al., 2009) and a higher risk of mortality by *S. aureus* bacteremia than men (Mansur et al., 2012); furthermore, estradiol levels were directly correlated with the severity of sepsis (Tsang et al., 2016).

It is well known that innate immunity is an organism’s first line of defense and monocytes and macrophages (MΦ) play an important role by eliminating invading pathogens through phagocytosis and producing reactive oxygen and nitrogen species, and also releasing various inflammatory mediators, including cytokines and chemokines, which are essential for initiating and spreading the inflammatory process (Marriott et al., 2006; Dimitrijević et al., 2013). The aim of this study was to assess the effect of E2 and menstrual cycle variations on monocyte/macrophage response induced by *S. aureus in vitro* using murine peritoneal macrophages (MPMs) and human peripheral blood monocytes (HPBMs).

## Methods

### Bacterial Strain

We used an *S. aureus* strain isolated from a hospital in a previous study by our group, which has norA, mecA and blaZ genes, respectively, resistant to fluoroquinolone and beta-lactams (Carvalho et al., 2017). We used Brain Heart Infusion (BHI) and mannitol agar to activate, cultivate and sub-cultivate *S. aureus* strains for further inoculation in MPMs and HPBMs. In addition, Gram stain, catalase test, coagulase test and PCR (nuc gene) (Sasaki et al., 2010) were performed to confirm the identity and purity of the bacterial strain.

### *S. aureus* Inoculum

An inoculum of *S. aureus* was prepared by direct suspension of 3 to 5 morphologically identical colonies of the reference strain into tubes containing sterile saline solution. The tubes were then vortexed and analyzed using a spectrophotometer to obtain an absorbance of 0.135 at 660 nm, equivalent to 1 × 10^8^ colony-forming units (CFU) (de Oliveira et al., 2015). MPMs and HPBMs were subsequently inoculated at a multiplicity of infection (MOI) of 1:100.

### *In Vitro* Model of Murine Peritoneal Macrophages (MPMs)

#### Mice

Twelve female and six male BALB/c Specific Pathogen Free (SPF) mice aged six to eight weeks were used. The mice were obtained from the Multidisciplinary Center for Biological Research in the Science Area of the Animal Laboratory of the State University of Campinas (CEMIB/UNICAMP) and were kept under controlled conditions of light (light from 7 am to 7 pm) and temperature (23 ± 3°C), with free access to water and feed in the bioterium for mice of the Multidisciplinary Health Institute of the Federal University of Bahia (IMS/UFBA).

All experiments with mice were conducted in accordance with internationally accepted principles for the use and care of laboratory animals, as established in the Brazilian guideline for the care and use of animals in teaching or scientific research activities (DBCA) related to principles of conduct that ensure the care and ethical management of animals used for scientific or teaching purposes, and carried out after approval by the Ethics Committee on the Use of Animals (CEUA) of the University of São Paulo, under protocol 03/2013.

#### Ovariectomy

Females were ovariectomized bilaterally, a model in which female sex hormones are significantly decreased (Dimitrijević et al., 2013), or submitted to sham operation. The animals were anesthetized with xylazine and ketamine at doses of 5 mg/kg and 50 mg/kg respectively (de Andrade et al., 2011). The surgery was carried out according to the current method in endocrinology (Mirbaha et al., 2009). The animals received antibiotic prophylaxis with 2.5% enrofloxacin in a single dose of 2.5 mg/kg. It is worth mentioning that the half-life of this antibiotic in mice is 89 minutes (Bregante et al., 1999)

#### MPM Isolation

To elicit peritoneal cells, 1 ml of 3% thioglycollate medium was injected into the peritoneal cavity of male mice (n = 6), OVX females (n = 6) and sham females (n = 6) (Layoun et al., 2015). After three days, the mice were euthanized by deepening anesthesia (xylazine 30 mg/kg and ketamine 300 mg/kg). The cells were isolated from peritoneal lavage according to the protocol described in the literature (Zhang et al., 2008; Lu et al., 2013) and cultured in RPMI 1640 medium (Gibco ™) supplemented with 10% Bovine Fetal Serum (SFB) (Sigma Aldrich) and ciprofloxacin (50 µg/ml) (Sigma Aldrich). The MPM suspension (2 x 10^5^/ml) was plated on 24-well plates.

#### Inoculation of MPMs With *S. aureus* and/or 17β-Estradiol

MPMs from OVX and sham female mice were cultured with the inoculum of *S. aureus* (1 x 10^6^ CFU –MOI = 1:100) or sterile saline solution for 6 hours in 5% CO_2_ and 95% humid air at 37 °C. Part of MPMs from OVX females were pre-treated with 17β-estradiol (10^-7^ M) (Sigma Aldrich) for 24 hours at 37°C before stimulation with *S. aureus*. To assess whether sex would influence the response of macrophages to *S. aureus*, we performed the same experiment with MPMs derived from male mice.

#### Evaluation of Relative Cytokine Gene Expression in Female and Male MPMs

After being removed with trypsin, the MPMs were stored with RNAlater at -70°C and, later, destined for the gene expression of inflammatory markers by the RT-qPCR array methodology. The MPMs mRNA was extracted using the TRIzol^®^ Plus RNA purification kit (Thermo Fisher Scientific, Waltham, MA, USA) following the protocol provided by the manufacturer. The cDNA was obtained by a SuperScript III Reverse Transcriptase Kit. We used personalized plates of the target genes IL-1β, IL-6, IL-8, TNF-α, and TLR2. The SYBR^®^ PCR Master Mix test gene expression (Applied Biosystems™) was performed on personalized plates with targets genes, endogenous glyceraldehyde 3-phosphate dehydrogenase (GAPDH) and β-actin genes and MGDC control genes (Mouse Genomic DNA Contamination), RTC (Reverse Control Transcription) and PPC (Positive PCR Control). Classically activated macrophages in *S. aureus* more commonly adopt TLR ligation signaling *via* MyD88 and NF-κB, generating TNF-α, IL-1β, IL-6, and IL-8 (Cole et al., 2014; Tarique et al., 2015). Classically activated macrophages also express high levels of TLR2 (Cole et al., 2014), which has been greatly implicated in host defense against *S. aureus* (Miller and Cho, 2011; Krishna and Miller, 2012). Amplification was performed on the StepOnePlusTM Software v2.3 thermocycler, with the following parameters for all genes: 95°C for 10 minutes, followed by 40 cycles at 95°C for 15 seconds and 60°C for 1 minute. The data were analyzed using the comparative method (2^ΔΔCt^) and normalization was performed based on GAPDH expression.

### *In Vitro* Model of Human Peripheral Blood Monocytes (HPBMs)

#### Subjects

Volunteers were previously evaluated in a screening consultation in which a questionnaire was applied. The questionnaire asked for date of birth, sex, medical history and habits such as alcohol consumption, tobacco consumption, and medication use. Women were asked about their gynecological status such as menstrual changes and use of contraceptive drugs. The exclusion criteria were individuals under 18 years of age, underweight, obese, diabetic, taking any medication, and women who gave birth in the last six months, who had gynecological disorders associated with hormonal changes (such as polycystic ovary syndrome, myoma, among others), irregular menstrual cycle, in hormonal treatment and/or use of hormonal contraceptives in the last three months before blood collection. Peripheral blood was collected from six healthy men and six women. Donor health was also assessed using blood count and serological markers (**Table S1**). In women, a collection was performed two days before the expected day of ovulation (periovulatory period), when the estradiol peak occurs. A second collection was carried out within the first two days of the follicular phase (menstruation), during which the estradiol concentration is lower. In both periods, the collection dates were stipulated according to the information collected about the menstrual cycle. All blood samples were kept between 6 and 10°C and destined to isolate human monocytes within two hours after collection. Part of the blood collected was used to measure estradiol, progesterone, follicle-stimulating hormone and luteinizing hormone by the chemiluminescence method using a Backman Coulter Unicell DxI 800 to confirm the phase of the menstrual cycle reported by the volunteer (**Figure S1**). The study was developed after approval by the Human Research Ethics Committee of the Federal University of Bahia, Multidisciplinary Institute for Health (CAAE: 51919515.5.0000.5556) and was carried out in accordance with The Code of Ethics of the World Medical Association (Declaration of Helsinki) for experiments involving humans.

#### Isolation of Human Peripheral Blood Monocytes (HPBMs)

The monocytes were separated by centrifugation in a Ficoll column (400 g for 20 min), washed and centrifuged with PBS (Phosphate Buffered Saline) 1x twice (100 g for 10 minutes) and resuspended in RPMI 1640 medium containing 10% serum fetal bovine, 2 Mm glutamine, 10 HEPES and 20 μl/ml ciprofloxacin. After evaluating the viability and adequate confluence (2 x 10^5^/ml), the cells were placed in 24-well polystyrene plates and incubated for 24 hours at 37°C with 5% CO_2_ before inoculation with *S. aureus* (Campesi et al., 2012; Santos-Junior et al., 2018). The monocytes adhere to the polystyrene surface (Kramer and Wray, 2002); thus, non-adherent cells (mainly lymphocytes) were washed away. Although primary monocytes are an excellent model for deciphering E2-induced changes in gene transcription, donor variability can limit the study of these cells (Teixeira et al., 2016), consequently, we performed the culture using six different donors of each sex.

#### Inoculation With *S. aureus* and Stimulation of HPBMs With 17β-Estradiol

The HPBMs were inoculated with *S. aureus* at an MOI of 1:100 or sterile saline for six hours in an incubator at 37°C with 5% CO_2_. Prior to inoculation, HPBMs from men were incubated with 17β-estradiol (E2) (Sigma Aldrich) for 24 hours at 37°C (10^-7^ M) (Aomatsu et al., 2013). Negative controls were performed by incubating cells without and with the E2 vehicle (absolute alcohol). All experiments were carried out in quintuplicate. The supernatant of each culture was collected and frozen at -70°C to measure cytokine production. For gene expression assays, cells were removed with trypsin and stored at -70°C with RNAlater (Invitrogen) in microtubes for subsequent RNA extraction.

#### Dosage of Cytokines

The cell culture supernatant was removed and stored at -70 °C and, later, destined for measuring cytokines by the Luminex xMAP^®^ immunoassay methodology using the ProcartaPlex Immunoassay kit (Affymetrix eBioscience) according to the manufacturer’s instructions. The following cytokines were measured: GM-CSF; TNF-α; IL-1β; IL-6; IL-10; IL-12; IL-18; IL-23 and IL-27.

#### Evaluation of Gene Expression

After removing with trypsin, the HPBMs were stored with RNAlater at -70 °C and, later, they were destined for gene expression. The gene expression of inflammatory markers was evaluated by RT-qPCR array methodology. The mRNA of the HPBMs samples was extracted using TRIzol^®^ LS (Life Technologies™) following the protocol provided by the manufacturer. The cDNA was obtained by means of a retro-transcription (RT) from the mRNA, using the SuperScript^®^ III Reverse Transcriptase kit with addition of oligonucleotides complementary to the poly-A tail of the mRNA, (Oligo dT) and inhibitor of RNAse. The cDNA obtained was subjected to analysis by the Human Innate & Adaptive Immune Responses PCR Array kits (Qiagen-SABioscience), for evaluating genes involved in the response of host macrophages associated with bacterial infection. The qPCR array kit allows for analyzing the expression of 84 genes involved in the host’s response to bacterial infection. This array includes genes related to TLR signaling, as well as genes involved in the acute phase response, activation of the complement system, inflammatory response and acquired immune response against bacteria. All procedures and data analysis were performed according to the manufacturer’s instructions and software Qiagen-SABioscience (https://dataanalysis.qiagen.com/pcr/arrayanalysis.php).

### Statistical Analysis

Statistical analysis was performed using the GraphPad-Prism 5.0 program (GraphPad Softwear, San Diego, CA-USA). The comparisons made in the different experiments were determined by individual variation or error variation (s2), through non-parametric analysis Mann-Whitney one-tailed test after performing the Shapiro-Wilk test to assess the normality of the data. The results were expressed as the mean plus or minus the standard deviation from the mean (SDM). Statistical differences were considered significant when *P* < 0.05 using a 95% confidence interval.

## Results

### *In Vitro* Model of Murine Peritoneal Macrophages (MPMs)

#### MPMs from Ovariectomized Females Express More TNF, IL-1 and IL-8

**Figure 1:**
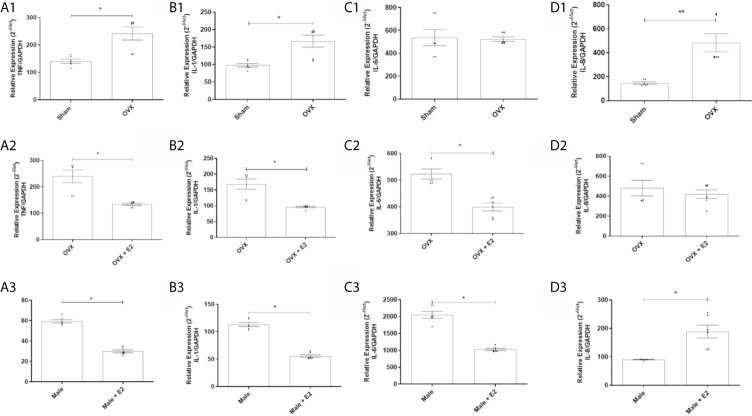
(1) Relative gene expression of the cytokines TNF **(A1)**, IL-1 **(B1)**, IL-6 **(C1)** and IL-8 **(D1)** of MPMs (2 x 10^5^/ml) from sham (n = 6) and OVX females (n = 6) inoculated with *S. aureus* (10^8^ CFU) for 6 h. (2) Relative gene expression of the cytokines TNF **(A2)**, IL-1 **(B2)**, IL-6 **(C2)** and IL-8 **(D2)** of MPMs (2 x 10^5^/ml) from OVX females (n = 6) stimulated or not with 17β-estradiol (E2) (10^-7^ M) for 24 h before inoculation with *S. aureus* (10^8^ CFU) for 6 h. (3) Relative gene expression of cytokines TNF **(A3)**, IL-1 **(B3)**, IL-6 **(C3)** and IL-8 **(D3)** of MPMs (2 x 10^5^/ml) of males (n = 6) stimulated or not with 17β-estradiol (E2) (10^-7^ M) for 24 h before inoculated with *S. aureus* (10^8^ CFU) for 6 h. Evaluated by RT-qPCR array. Data expressed as mean ± SDM. *P < 0.05. **P < 0.01.

It was observed that MPMs from ovariectomized (OVX) females showed a significantly higher relative gene expression of the pro-inflammatory cytokine TNF (**Figure 1A1**) and IL-1 (**Figure 1B1**) and the chemokine IL-8 (**Figure 1D1**) compared to MPMs from sham females. However, no difference was observed between groups when analyzing the cytokine IL-6 (**Figure 1C1**).

#### 17β-Estradiol Reduces Relative Gene Expression of Pro-Inflammatory Cytokines in MPMs of Ovariectomized Females

Treatment with 17β-estradiol in MPMs from OVX females significantly reduced relative gene expression of cytokines TNF (**Figure 1A2**), IL-1 (**Figure 1B2**) and IL-6 (**Figure 1C2**) compared to MPMs from untreated OVX females. However, a significant difference induced by treatment with E2 in the expression of IL-8 was not observed (**Figure 1D2**).

#### 17β-Estradiol Reduces Relative Gene Expression of Pro-Inflammatory Cytokines in MPMs of Males

E2 treatment induced a significant reduction in the gene expression of cytokines TNF (**Figure 1A3**), IL-1 (**Figure 1B3**) and IL-6 (**Figure 1C3**) in MPMs compared to MPMs of untreated males. However, a significant increase in IL-8 expression was observed in MPMs from males treated with E2 compared to MPMs from untreated males (**Figure 1D3**).

#### MPMs From Ovariectomized Females and Males Express More TLR2, but 17β-Estradiol Reduced This Expression

**Figure 2:**
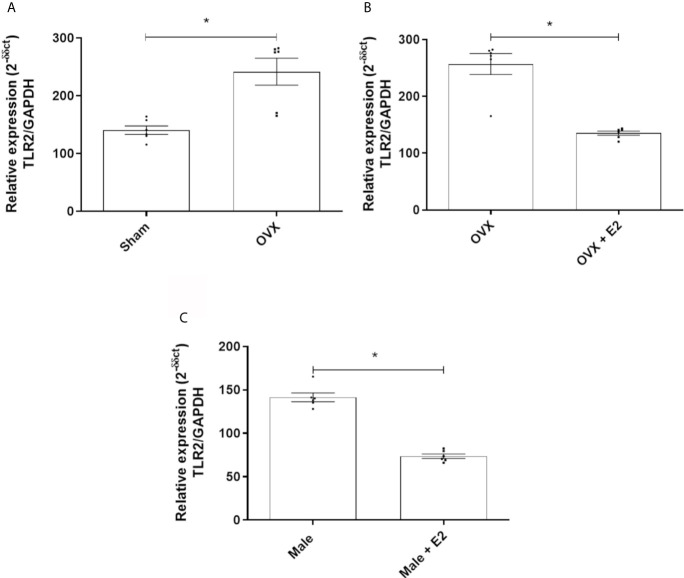
**(A)** Relative gene expression of TLR2 of MPMs (2 x 10^5^/ml) from sham (n = 6) and OVX females inoculated with *S. aureus* (10^8^ CFU) for 6 h. **(B)** Relative gene expression of TLR2 from OVX females (n = 6) stimulated or not with 17b-estradiol (E2) (10^-7^ M) for 24 h before inoculation with *S. aureus* (10^8^ CFU) for 6 h. **(C)** Relative gene expression of TLR2 of MPMs (2 x 10^5^/ml) of males (n = 6) stimulated or not with 17b-estradiol (E2) (10^-7^ M) for 24 h before inoculation with *S. aureus* (10^8^ CFU) for 6 h. Evaluated by RT-qPCR array. Data expressed as mean ± SDM. *P < 0.05.

MPMs of OVX females express significantly more TLR2 receptors than sham female MPMs (**Figure 2A**). Treatment with E2 significantly reduced expression of this receptor in MPMs from OVX females (**Figure 2B**) and in MPMs from males (**Figure 2C**).

### *In Vitro* Model of Human Peripheral Monocytes (HPBMs)

#### 17β-Estradiol Reduces the Production of IL-1β, IL-6, TNF-α and GM-CSF by HPBMs of Men, and HPBMs of Women Produce Less IL-1β and TNF-α Than HPBMs of Men

**Figure 3:**
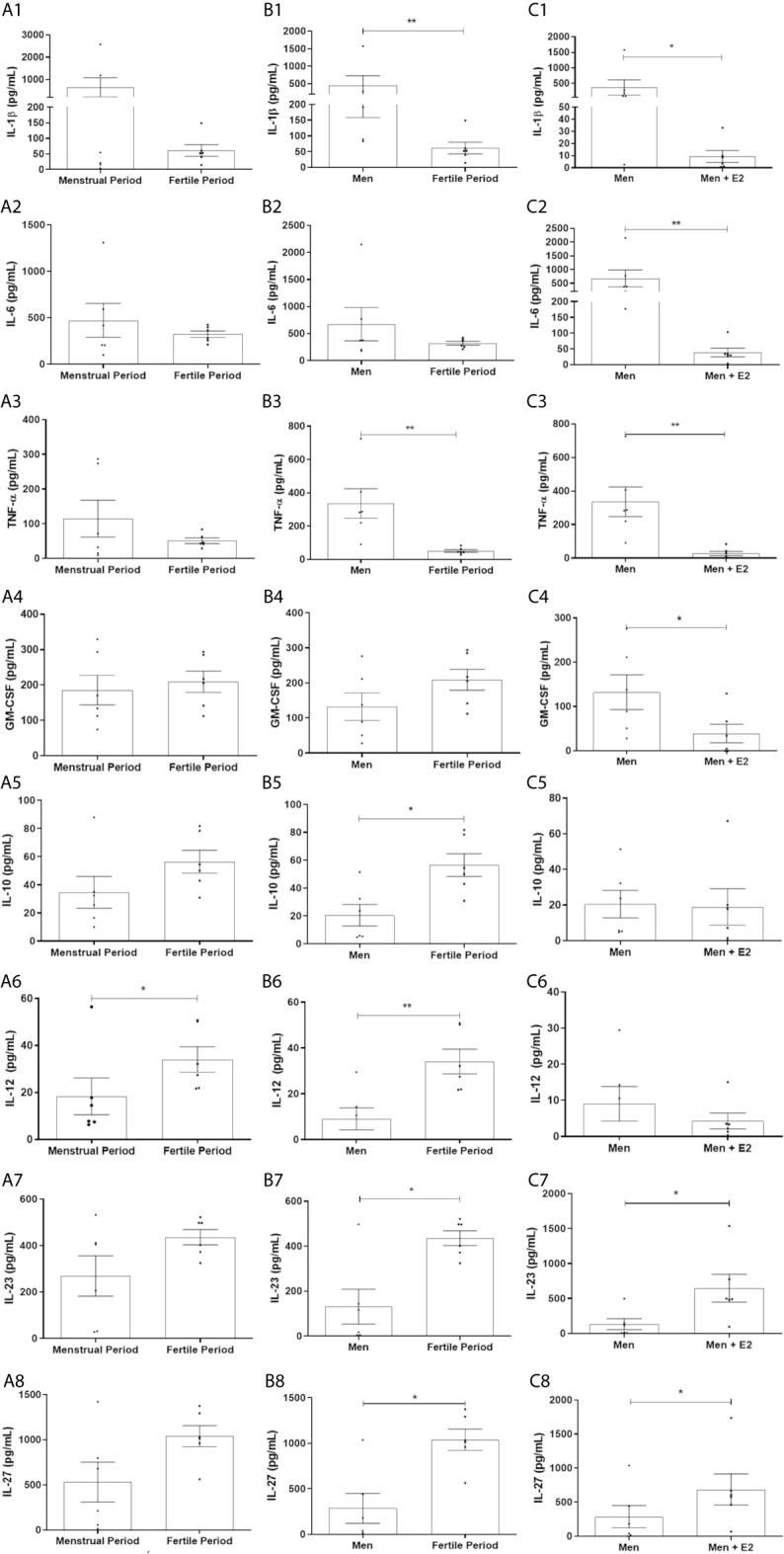
Concentration of IL-1β, IL-6, TNF-α, GM-CSF, IL-10, IL-12, IL-23 and IL-27 in the supernatant of HPBMs of men and women inoculated with *S. aureus* (10^8^ CFU). HPBMs of women in the first two days of their menstrual period or in the periovulatory period inoculated with *S. aureus* **(A1–A8)**; HPBMs of men or women in the periovulatory period inoculated with *S. aureus* **(B1–B8)**; HPBMs from men inoculated with *S. aureus* or men inoculated with *S. aureus* and previously treated with 17β-estradiol (E2) (10^-7^ M) **(C1–C8)**. Evaluated by Luminex. Data expressed as mean ± SDM. *P < 0.05. **P < 0.01.

Although no significant difference was observed between HPBMs of women during the first two days of their menstrual period and women during their fertile period (periovulatory) (**Figures 3A1–A4**), the secretion of IL-1β and TNF-α by HPBMs of women during their fertile period was significantly less than the secretion by HPBMs of men (**Figures 3B1–B4**). In addition, HPBMs from men pretreated with E2 showed significantly reduced secretion of IL-1β, IL-6, TNF-α and GM-CSF compared to untreated HPBMs from men (**Figures 3C1–C4**).

#### 17β-Estradiol Increases the Production of IL-23 and IL-27 by HPBMs of Men, and HPBMs of Women Produce More IL-10, IL-12, IL-23 and IL-27 Than HPBMs of Men

HPBMs of women during their fertile period produced more IL-12 than HPBMs of women during their menstrual period (**Figure 3A6**). Still, HPBMs of women during their fertile period produced significantly more IL-10, IL-12, IL-23, and IL-27 compared to HPBMs of men (**Figures 3B5–B7**) and pretreatment with 17B-estradiol significantly increased production of IL-23 and IL-27 by HPBMs of men (**Figures 3C7, B8**).

#### Comparison of Gene Expression of HPBMs (Men and Women) Inoculated With *S. aureus* and Inoculated With Sterile Saline

Among the 84 genes analyzed, a statistically significant difference was observed in 37 genes with positive regulation in HPBMs inoculated with *S. aureus* compared to HPBMs inoculated with sterile saline. The genes regulated positively (*P* < 0.05) were BTK, CCL2, CD14, CD180, CD80, CD86, CHUK, ECSIT, IL12A, IL1A, IL1B, IL6, IRAK2, IRF1, MAP2K3, MAP2K4, MAP3K1, MAP3K7, MAP4K4, MAPK8, MAPK8IP3, NFKB2, NFKB1, PTGS2, RELA, RIPK2, SARM1, SIGIRR, TAB1, TBK1, TICAM1, TIRAP, TLR2, TLR9, TNF and TNFRSF1A (**Figure S2**).

#### Comparison of the Gene Expression of the HPBMs of Women During Their Menstrual Period With the HPBMs of Women During Their Fertile Period

**Figure 4:**
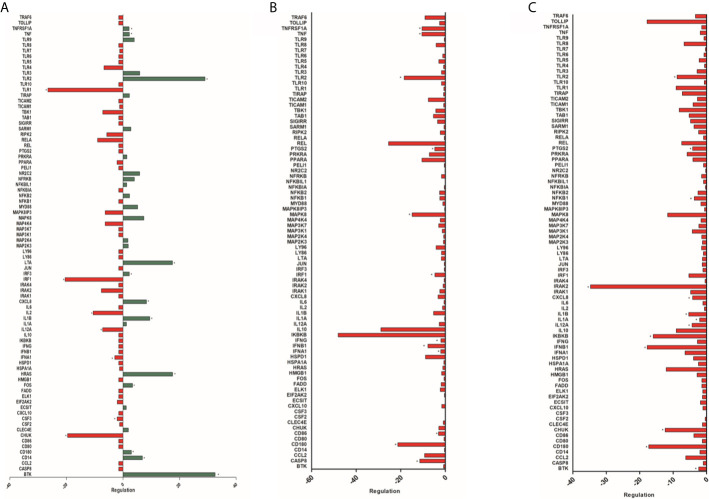
**(A)** Analysis of 84 genes involved in the host’s response to bacterial infections of HPBMs inoculated with *S. aureus* from women during their menstrual period compared to HPBMs from women during their fertile period. Analysis performed with the Human Innate & Adaptive Immune Responses PCR Array kit (Qiagen - SABioscience). Positively regulated genes (green) and negatively regulated genes (red). *P < 0.05 *vs.* HPBMs inoculated with *S. aureus* from women during their fertile period. **(B)** Analysis of 84 genes involved in the host’s response to bacterial infections of HPBMs inoculated with *S. aureus* from women during their fertile period compared to HPBMs from men. Analysis performed with the Human Innate & Adaptive Immune Responses PCR Array kit (Qiagen - SABioscience). Negatively regulated genes (red). *P < 0.05 *vs.* HPBMs inoculated with *S. aureus* from men. **(C)** Analysis of 84 genes involved in the host’s response to bacterial infections of HPBMs inoculated with *S. aureus* from men treated with E2 compared to HPBMs from men. Analyzed by the Human Innate & Adaptive Immune Responses PCR Array kit (Qiagen - SABioscience). Negatively regulated genes (red). Data expressed as mean ± SDM. *P < 0.05 *vs.* HPBMs inoculated with *S. aureus* from men.

Among the 84 genes analyzed, a statistically significant difference (*P* < 0.05) was observed in 12 genes with positive regulation - TNFRSF1A, TNF, TLR2, LTA, IRF3, CXCL8, IL1B, HRAS, FOS, CD180, CD14, BTK - and seven negatively regulated genes - TLR1, IRF1, IL2, IL12A, IFNA1, CSF3, CHUK - in HPBMs inoculated with *S. aureus* in women during their menstrual period compared to HPBMs in women during their fertile period (**Figure 4A**).

#### Comparison of Gene Expression of HPBMs of Women During Their Fertile Period and HPBMs of Men

Among the 84 genes analyzed, a statistically significant difference (*P* < 0.05) was observed in 12 genes with negative regulation in the HPBMs inoculated with *S. aureus* of women during their fertile period compared to the HPBMs of men. They were TNFSF1A, TNF, TLR2, PTGS2, MAPK8, IRF1, IFNG, IFNB1, IFNA1, CD86, CD180 and CASP8. However, no positively regulated genes were observed HPBMs of women during their fertile period compared to HPBMs of men (**Figure 4B**).

#### Comparison of Gene Expression of HPBMs from Men and HPBMs From Men Treated With E2

Among the 84 genes analyzed, a statistically significant difference (*P* < 0.05) was observed in 13 genes with negative regulation in the HPBMs inoculated with *S. aureus* of men treated with E2 compared to the HPBMs of men. They were TLR2, PTGS2, NFKB1, IRAK2, CXCL8, IL1B, IL1A, IL12A, IKBKB, IFNB1, CHUK, CD180 and BTK. However, no positively regulated genes were observed in the HPBMs of men treated with E2 compared to the HPBMs of men not treated (**Figure 4C**).

## Discussion

The results of the present study provide evidence in support of the anti-inflammatory effects of Estrogen in innate immunity. Using HPBMs from male and female healthy donors, and MPMs from male, sham, and OVX female mice, we have shown that MPMs from female sham exhibit lower gene expression of TNF-α than those from OVX females. Supporting our hypothesis, TNF-α expression was significantly reduced when female OVX MPMs were treated with E2; a reduction was also observed in treated male MPMs. In addition, HPBMs of women during their fertile period, compared to HPBMs of men, showed lower expression of TNF and TNFRSF1A (a membrane receptor for TNF-α) (Oertelt-Prigione, 2012). HPBMs of women during their fertile period and E2-treated HPBMs of men also showed lower production of TNF-α than untreated HPBMs of men. Consistent with our results, Santos et al. (2017) and Margaryan et al. (2017) reported that E2 inhibited LPS-induced TNF-α production in embryonic fibroblast cells (Santos et al., 2017) and whole blood cell culture (Margaryan et al., 2017). However, not all studies in the literature support this observation. Dong et al. (2015) showed that the expression and production of TNF-α in peripheral blood mononuclear cells (PBMCs) from postmenopausal women were lower than that of PBMCs from premenopausal women. In the same study, OVX female mice were reported to have lower serum levels of TNF-α compared to female sham. The researchers also observed that E2 treatment in OVX females increased the serum concentration of this cytokine. Khan et al. (2015) observed that treatment with E2 increased the production of TNF-α in peritoneal MΦ of women compared to that in untreated MΦ. These differences show that the effects of E2 on cytokine secretion depend on the cell type and Estrogen concentration. It is likely that the stimulatory effects of E2 on TNF secretion are exerted in lower levels of E2, whereas high E2 levels during pregnancy result in inhibition of TNF secretion (Straub, 2007).

MPMs of female sham showed lower IL-1β gene expression than those of OVX females. E2 treatment reduced the expression of IL-1β in the MPMs of both OVX females and males. In addition, male HPBMs treated with E2, and female HPBMs during the fertile period showed lower expression of this cytokine compared to untreated male HPBMs. Zhang et al. (2008) also observed an attenuation of the inflammatory response in E2 treated ATCC RAW 264.7 culture cells through reduction in the expression and production of IL-1β. However, in contrast to our findings, Stanojević et al. (2015) reported that LPS stimulation results in lower production of IL-1β and TNF-α in MPMs of OVX females compared to those in sham, whereas Teixeira et al. (2016) found no difference in the production of IL-1β in E2 treated monocyte-derived macrophages (M1) from postmenopausal women versus untreated M1s. In another study, MPMs of middle-aged female rats, with significantly reduced levels of circulating estradiol, were reported to have a lower concentration of IL-1β compared to MPMs of young females after stimulation with LPS (Ćuruvija et al., 2017). Calippe et al. (2010) demonstrated that MPMs from OVX females treated with E2 and female sham have higher expression and production of IL-1β, along with IL-6, compared to MPMs from untreated OVX females. However, it is worth mentioning that their study comprised of chronic E2 administration *in vivo*, while the results of our study reflect the effect of short-term *in vitro* hormonal exposure with a high dose of E2 (pregnancy levels) (Straub, 2007).

There was no significant difference observed in the expression of IL-6 between MPMs from OVX and female sham; however, Estrogenic stimulation caused a reduction in the expression of IL-6 in MPMs from both OVX females and males. Furthermore, E2 treatment significantly reduced the production of IL-6 in male HPBMs. In accordance with our results, Pelekanou et al. (2016) reported that pre-treatment of HPBMs with E2 for 24 h significantly inhibited the inflammatory response of IL-6 induced by LPS stimulation. Estrogen also inhibited the secretion of IL-6 by Kupffer cells, (Naugler et al., 2007) and a brief exposure to E2 for 1 h significantly attenuated the production of IL-6 by embryonic fibroblast cells, in both males and females, after stimulation with LPS (Santos et al., 2017). Liu et al. (2014) also observed attenuated production of IL-6 in bone marrow-derived macrophages, mediated by previous exposure to E2 for 24 h.

The chemokine IL-8 (also known as CXCL8) contributes to the recruitment and migration of neutrophils and monocytes by activating integrins (Rao et al., 2007). In our study, we observed that female sham MPMs showed a lower expression of this chemokine compared to MPMs in OVX females. HPBMs of women during their fertile period expressed less CXCL8 than HPBMs of women during their menstrual period, while E2 treatment reduced the expression of IL-8 in HPBMs of men. However, contrary to what was observed in human phagocytes, the male MPMs treated with E2 showed a significant increase in the expression of this chemokine. The literature available on estrogenic influence of monocytes/macrophages on IL-8 production is scarce and controversial. Margaryan et al. (2017) did not report a change in IL-8 production in whole blood cells after LPS stimulation and E2 treatment whereas Shi et al. (2006) found that E2 inhibited the secretion of IL-8 by U-937 cells (a monocyte cell line). Rodríguez et al. (2002) similarly reported that E2 inhibited the secretion of IL-8 in HUVEC cells.

Although the production of the anti-inflammatory cytokine IL-10 is mostly associated with M2 macrophages, M1 macrophages are also known to produce low levels of this cytokine (Shapouri-Moghaddam et al., 2018). In this study, it was observed that HPBMs of women during their fertile period produced more IL-10 compared to HPBMs of men. Teixeira et al. (2016) observed that treatment with E2 increased the production of IL-10 in HPBMs of postmenopausal women. Levels of IL-10 mRNA in E2 treated MPMs from OVX females were significantly higher than those in MPMs from female sham and untreated OVX mice (Pepe et al., 2017). Pomeroy et al. (2016) observed that bovine monocyte-derived dendritic cells (mDCs), stimulated with *E. coli* and treated with E2 and progesterone (PG) together, exhibited increased IL-10 production. However, treatment with either E2 or PG alone did not induce upregulation of IL-10. Galván-Ramírez et al. (2019) reported that THP-1 monocytes, stimulated with *Toxoplasma gondii* and treated with E2 showed increased production of IL-10; however, no differences were observed when pre-treated with progesterone. Secretion of IL-10 was suppressed by E2 in a dose-dependent manner in NR8383 macrophages (Kou et al., 2015). Such contrasting results might be due to the diverse effects of E2 on different subtypes of immune cells (Straub, 2007).

The HPBMs of women during their menstrual period exhibited downregulation of IL-12 than the HPBMs of women during their fertile period. However, it was observed that E2 treated HPBMs of men did not show increased production of this cytokine; and the IL-12 gene expression in these cells was lower compared to untreated HPBMs. Contrary to our observations, Galván-Ramírez et al. (2019) demonstrated that THP-1 monocytes, pre-treated with E2, and subsequently stimulated with *T. gondii* cells, exhibit downregulation of IL-12. However, their study differed in terms of the pre-treatment duration, with pre-treatment lasting for 48 h in Galván-Ramírez et al. (2019), whereas in our study, pre-treatment lasted for 24 h. In a study with LPS-stimulated whole blood culture, it was observed that during the pre-ovulatory phase, estradiol significantly increased the levels of IL-12 production, while E2 significantly decreased IL-12 levels during pregnancy (Matalka, 2003).

Another salient finding of this study is that the macrophages of OVX females were observed to express more TLR2 than the female sham. Treatment with E2 decreased the expression of this receptor in macrophages of OVX females and males. The HPBMs of women during their menstrual period expressed more TLR2 than those of women during their fertile period, which corroborates with our findings in murine macrophages. In addition, HPBMs of men showed higher expression of this receptor, compared to the HPBMs of women during their fertile period and E2 treated HPBMs of men. Giannoni et al. (2011) reported that estradiol decreases the innate immune response of mononuclear cells in the umbilical cord by decreasing the expression of TLR2. The findings of our study align with this observation. Further corroboration is provided by Jitprasertwong et al. (2016) demonstrating a significant decrease in the expression of TLR2 in monocytes stimulated with estradiol. On the contrary, Soucy et al. (2005) reported ovariectomy as the reason behind reduced expression of TLR2 in the brain after LPS stimulation, compared to the expression in healthy females. Rettew et al. (2009) point to a different effect mediated by E2 in another member of the TLR family, TLR4. The researchers showed that ovariectomy decreased the expression of TLR4 in murine macrophages, and that the exogenous *in vivo* replacement of E2, but not progesterone, elevated the expression of this receptor in macrophages. In view of these accounts, our data suggest a microorganism-specific effect mediated by E2.

Our study showed that HPBMs inoculated with *S. aureus* express more TLR9 than HPBMs inoculated with sterile saline alone. TLR9 is described as an intracellular receptor (Heftrig et al., 2017), but there are studies describing its expression on the cell surface of monocytes as well (Eaton-Bassiri et al., 2004; Saikh et al., 2004). Nurjadi et al. (2018), and Parker & Prince (2012) identified TLR9 as a critical receptor that mediates the induction of type I IFN signaling in dendritic cells in response to *S. aureus*, illustrating an additional mechanism through which *S. aureus* exploits innate immune signalling (Parcina et al., 2008; Parker and Prince, 2012). We found that HPBMs of women during their fertile period exhibit lower expression of the type I IFN coding genes IFNA1 and IFNB1, compared to HPBMs of men. In addition, HPBMs of these women downregulate IFNG, coding for IFN-γ and IRF1, which regulate the expression of target genes by binding to an interferon-stimulated response element (ISRE) in their promoters (Zenke et al., 2018). This negative regulation of IFNB1 was also observed in E2 treated HPBMs of men, compared to untreated HPBMs.

Furthermore, the expression of CD86 gene encoding the B7-2 co-stimulator protein involved in antigen presentation, is lower in HPBMs of women during their fertile period, compared to HPBMs in men (Re et al., 2002). The expression of CD180, a protein present on the surface of antigen-presenting cells (APCs), belonging to the TLR family, and PTGS2, encoding for cyclooxygenase 2 (COX-2) enzyme, is also reduced in HPBMs of females compared to males (Imayoshi et al., 2006; Kunzmann et al., 2013). This decreased expression of PTGS2 was also observed in E2 treated HPBMs of men compared to untreated HPBMs. The HPBMs in women showed negative regulation of CASP8 and MAPK8 transcription factors involved in TNF-induced apoptosis signalling pathway, compared to HPBMs in men (Peteranderl and Herold, 2017). However, further studies are needed to elucidate the effects of E2 on antigen presentation and cell apoptosis pathways.

Monocyte activation is necessary for the initiation and modulation of the immune response, mainly through the NF-κB pathway, that leads to the production and secretion of pro-inflammatory mediators and cytokines (Giannoni et al., 2011; Pelekanou et al., 2016). In the present study, E2 treated HPBMs of men exhibited lower expression of NF-κB1, compared to untreated HPBMs. However, no significant difference was observed with respect to MyD88, an adaptor molecule that is critical for TLR signalling. Giannoni et al. (2011) also demonstrated that estradiol mediates its anti-inflammatory activity, not through MyD88, but by inhibiting the NF-κB pathway. Santos et al. (2017) also demonstrated that E2 attenuated LPS-induced inflammation by suppressing the NF-κB-p65 axis in embryonic fibroblasts. Additionally, our study showed that E2 is able to downregulate the BTK genes, involved in the functioning of monocytes (Cavaliere et al., 2017) and IRAK2, encoding a kinase modulating the interleukin-1 receptor (IL1R) after stimulation. It has been reported that IRAK2 participates in the IL1 induced positive regulation of NF-κB pathway, in addition to the CHUK and IKBKB genes, which encode proteins that phosphorylate the inhibitor of the NF-κB complex.

In conclusion, this study proposes that 17β-estradiol inhibits the NF-κB pathway during the immune response in monocytes against *S. aureus* (**Figure S3**). Our findings provide new insights into the functioning of 17β-estradiol as an immunomodulator of *S. aureus* infection. This study shows that 17β-estradiol expression is associated with – (i) a decrease in pro-inflammatory cytokines such as TNF-α, IL-1β, IL-6, and GM-CSF, (ii) an increase in anti-inflammatory cytokines IL-10 and IL-27, (iii) suppression of TLR2 expression, and (iv) inhibition of the NF-κB pathway, suggesting an anti-inflammatory effect of 17β-estradiol during the immune response against *S. aureus*.

## Data Availability Statement

The raw data supporting the conclusions of this article will be made available by the authors, without undue reservation.

## Ethics Statement

The study also was developed after approval by the Human Research Ethics Committee of the Federal University of Bahia, Multidisciplinary Institute for Health (CAAE: 51919515.5.0000.5556). The patients/participants provided their written informed consent to participate in this study. The animal study was reviewed and approved by Ethics Committee on the Use of Animals (CEUA) of the University of São Paulo, under protocol 03/2013.

## Author Contributions 

Conceived and designed the experiments: CS, GC, JT, TS, and LM. *In vitro* Model of Murine Peritoneal Macrophages assays: CS, CB, HC, MJ, EN, MO, and EC. *In vitro* Model of Human Peripheral Blood Monocytes assays: CS, CB, HC, MJ, MT, DS, and RB. Analyzed the data: CS, CB, HC, TS, MO, GC, and LM. Contributed reagents/materials/analysis tools: JT, GC, and LM. Wrote the paper: CS, MO, TS, JT, GC, and LM. All authors contributed to the article and approved the submitted version.

## Funding

This study was supported by Fundação de Amparo à Pesquisa do Estado da Bahia (BOL0824/2011), Programa de apoio a pesquisadores emergentes da UFBA (PRODOC 02/2011) and Coordenação de Aperfeiçoamento de Pessoal de Nível Superior (Code 001).

## Conflict of Interest

The authors declare that the research was conducted in the absence of any commercial or financial relationships that could be construed as a potential conflict of interest.
