# Supplementary material for: Effects of 17β-Estradiol on Monocyte/Macrophage Response to Staphylococcus aureus: An In Vitro Study
Source: Front Cell Infect Microbiol. 2021 Jul 14;11:701391. doi: 10.3389/fcimb.2021.701391 (PMC8317603; doi:10.3389/fcimb.2021.701391)
Supplement: Supplementary file 1 [file DataSheet_1.docx]

**Table S1.** Evaluation of the general health status of the volunteers.

| **Evaluation of the General Health Status of the Volunteers** | | | | | |
| --- | --- | --- | --- | --- | --- |
|  | **Men**  *(n=6)* | **Reference value** | **Women**  *(n=6)* | **Reference value** | **p-value** |
| **Erythrocytes (millions/mm3)** | 5.50±0.30 | 4.5 to 5.9 | 4.94±0.12 | 4.5 to 5.9 | 0.0025** |
| **Leukocytes (/mm3)** | 5994.67±1,038.33 | 4000 to 10000 | 8418.44±1261.11 | 4000 to 10000 | 0.1970 |
| **Platelets** | 228400.00±31266.67 | 150000 to 50000 | 266838.89±35222.22 | 150000 to 450000 | 0.3496 |
| **Glucose (mg/dL)** | 80.00±4.33 | 60 to 99 | 84.33±6.50 | 60 to 99 | 0.5000 |
| **Total cholesterol (mg/dL)** | 183.33±19.56 | < 200 | 157.67±17.67 | < 200 | 0.0076** |
| **Triglycerides (mg/dL)** | 103.67±48.56 | < 150 | 66.00±8.75 | < 150 | 0.0571 |
| **HDL (mg/dL)** | 52.17±7.22 | 30 to 70 | 53.67±5.00 | 30 to 85 | 0.3149 |
| **VLDL (mg/dL)** | 20.83±9.78 | < 30 | 13.50±1.75 | < 30 | 0.0538 |
| **LDL (mg/dL)** | 110.33±13.56 | 100 to 129 | 92.11±12.78 | 100 to 129 | 0.0081** |
| **Creatinine (mg/dL)** | 0.77±0.10 | 0.6 to 1.3 | 0.67±0.06 | 0.6 to 1.3 | 0.0230* |
| **AST (U/L)** | 24.00±5.00 | < 50 | 20.39±2.22 | < 35 | 0.0378* |
| **Albumin (g/dL)** | 4.52±0.24 | 3.5 to 5.2 | 4.60±0.20 | 3.5 to 5.2 | 0.2598 |
| **Total Bilirubin (mg/dL)** | 0.70±0.17 | 0.3 – 1.2 | 1.20±0.40 | 0.3 – 1.2 | 0.4678 |
| **ALT (U/L)** | 30.67±18.56 | 7 to 52 | 16.78±2.44 | 7 to 52 | 0.1888 |
| **TSH (μUI/mL)** | 1.04±0.28 | 0.34 to 5.60 | 2.89±0.98 | 0.34 – 5.60 | 0.0465* |

Data are expressed as mean ± SDM. * p <0.05. ** p <0.01.


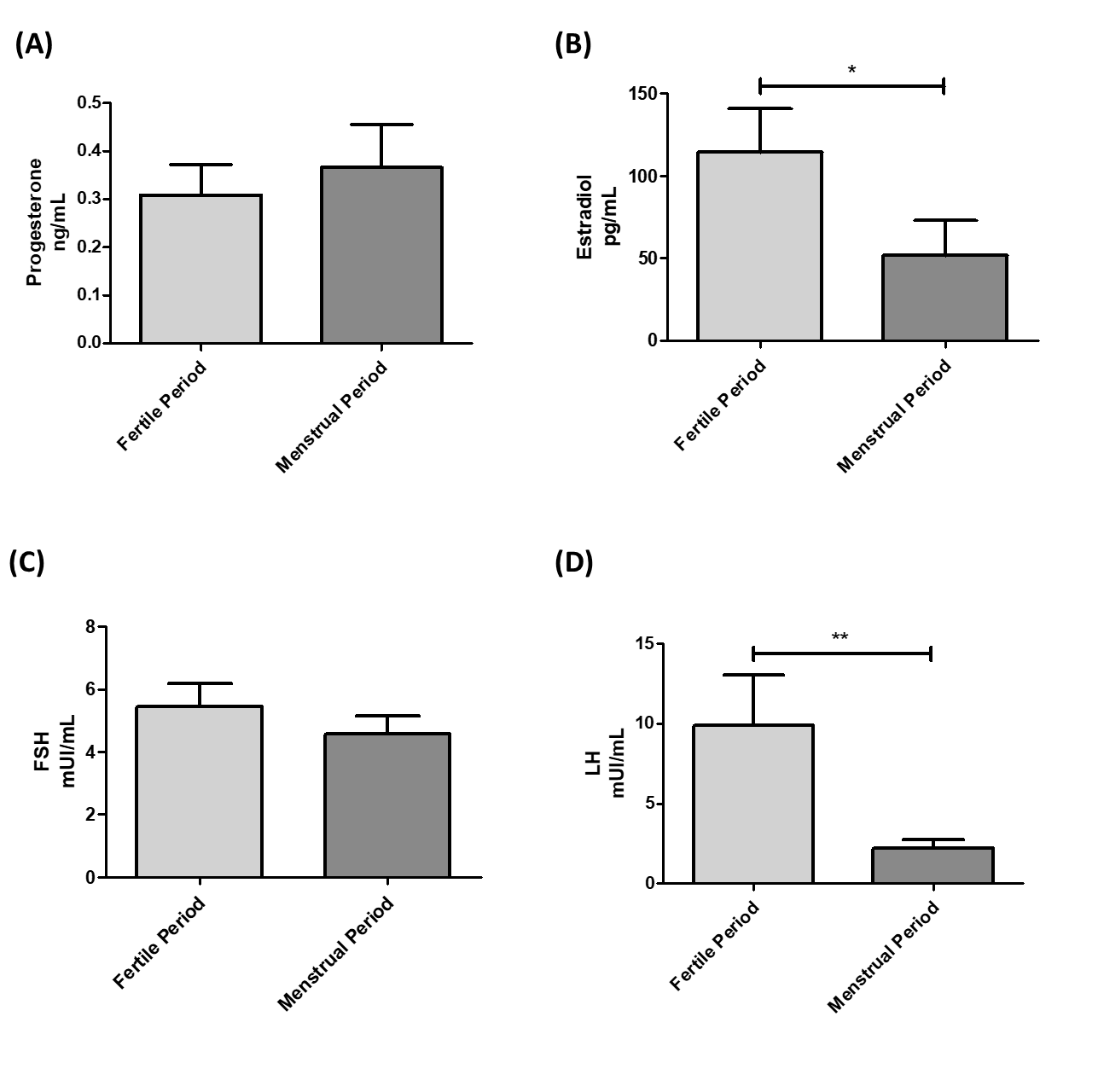


**Figure S1.** (A) Serum progesterone concentration in women in the periovulatory period and women in the first two days of the menstrual period. (B) Serum estradiol concentration in women in the periovulatory period and women in the first two days of the menstrual period. (C) Serum FSH concentration in women in the periovulatory period and women in the first two days of the menstrual period. (D) Serum LH concentration in women in the periovulatory period and women in the first two days of the menstrual period. Data are expressed as mean ± SDM. * p <0.05 vs Women (Menstrual Period).


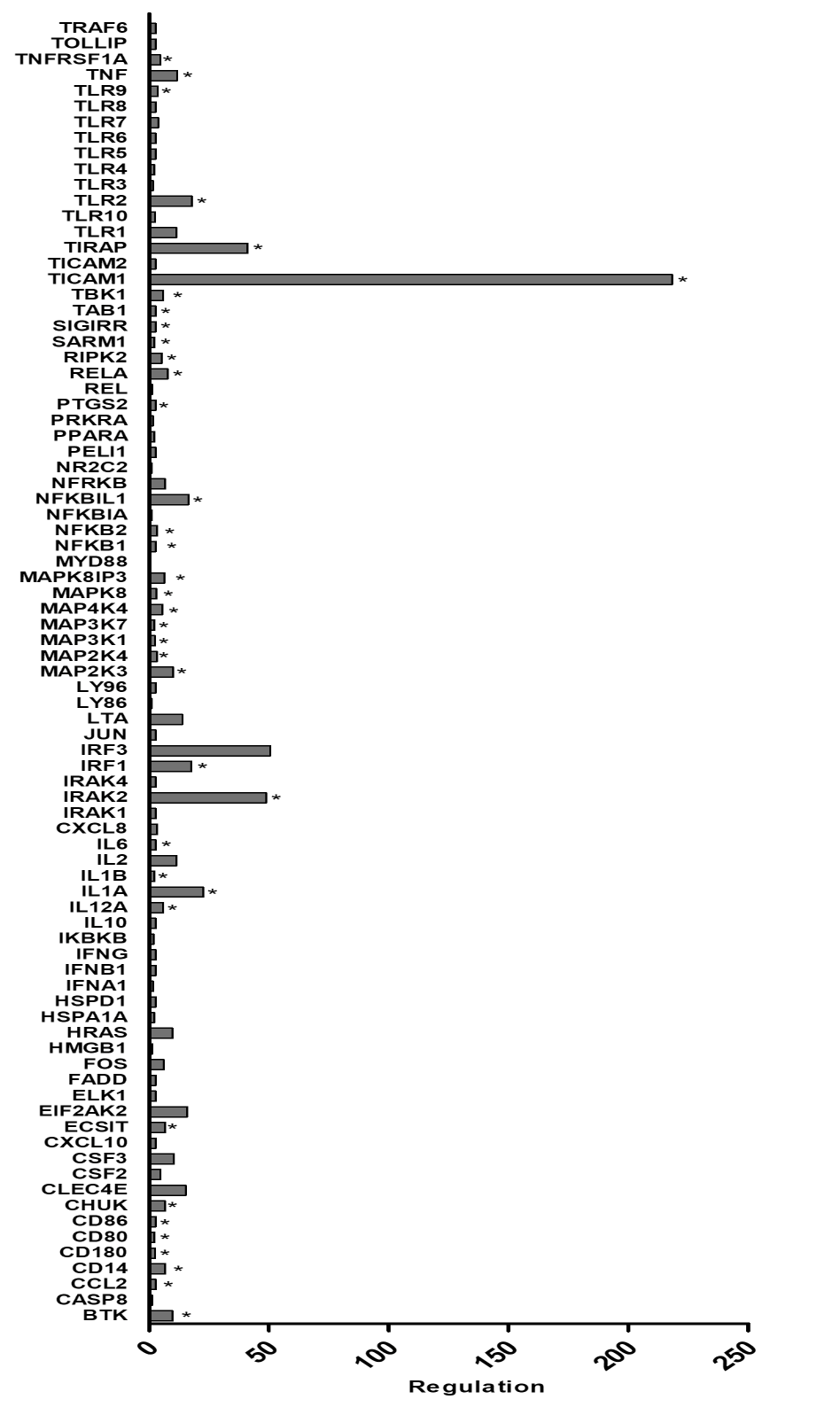


**Figure S2.** Analysis of 84 genes involved in the host's response to bacterial infections of HPBMs inoculated with *S. aureus* (10^8^ CFU) compared to HPBMs inoculated with sterile saline. Analysis performed with the Human Innate & Adaptive Immune Responses PCR Array kit (Qiagen - SABioscience). Positively regulated genes. Data expressed as mean ± SDM. * *P* < 0.05 vs HPBMs inoculated with sterile saline.


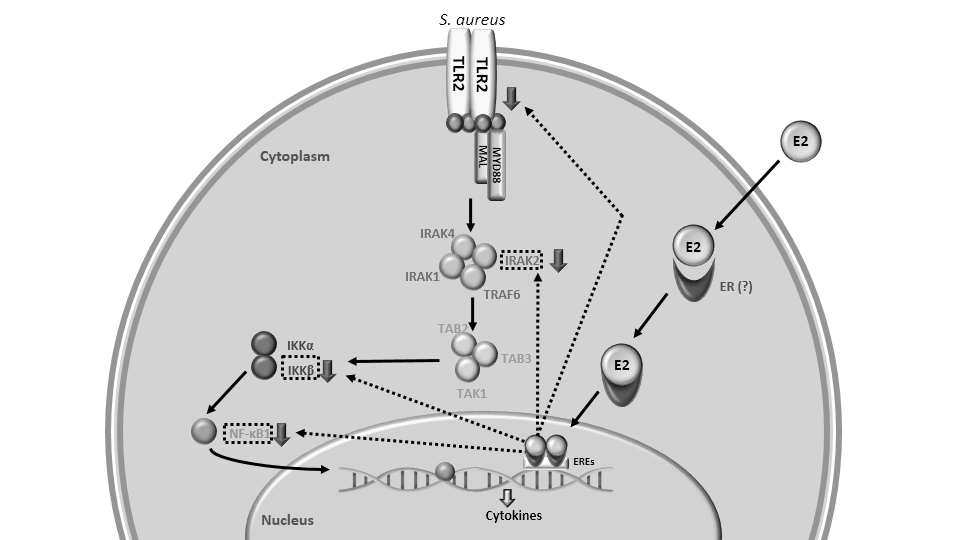


**Figure S3.** Effect of 17β-estradiol on immune response induced by *Staphylococcus aureus*. When binding to EREs, E2 acts by decreasing expression of TLR2 and transcription factors IRAK2, IKKβ and NF-κB (represented by dotted lines and arrows).
